# Research and Therapeutic Approaches in Stem Cell Genome Editing by CRISPR Toolkit

**DOI:** 10.3390/molecules28041982

**Published:** 2023-02-20

**Authors:** Behrouz Mollashahi, Hamid Latifi-Navid, Iman Owliaee, Sara Shamdani, Georges Uzan, Saleh Jamehdor, Sina Naserian

**Affiliations:** 1 Department of Pharmacy and Biotechnology (FaBiT), University of Bologna, 40126 Bologna, Italy; 2 Department of Molecular Medicine, National Institute of Genetic Engineering and Biotechnology, Tehran 14965/161, Iran; 3 Department of Virology, Faculty of Medicine, Hamadan University of Medical Sciences, Hamedan 6517838636, Iran; 4 INSERM UMR-S-MD 1197, Hôpital Paul Brousse, Paris-Saclay University, 94807 Villejuif, France; 5 CellMedEx, 94100 Saint Maur Des Fossés, France

**Keywords:** CRISPR, genome editing, stem cells, cell therapy, bioinformatics

## Abstract

The most widely used genome editing toolkit is CRISPR (clustered regularly interspaced short palindromic repeats). It provides the possibility of replacing and modifying DNA and RNA nucleotides. Furthermore, with advancements in biological technology, inhibition and activation of the transcription of specific gene(s) has become possible. Bioinformatics tools that target the evolution of CRISPR-associated protein 9 (Cas9) turn this protein into a vehicle that is specific for a DNA or RNA region with single guide RNA (sgRNA). This toolkit could be used by researchers to investigate the function of stem cell gene(s). Here, in this review article, we cover recent developments and applications of this technique in stem cells for research and clinical purposes and discuss different CRISPR/Cas technologies for knock-out, knock-in, activation, or inhibition of gene expression. Additionally, a comparison of several deliveries and off-target detecting strategies is discussed.

## 1. Introduction

Stem cells are heterogeneous and unspecialized cells that are the foundation of every organ and cell in our body [1]. Depending on their origin, stem cells are categorized into embryonic stem cells (ESCs), which exist in the inner cell mass (ICM) at an early stage of development; adult stem cells (ASC), which are found in specific tissues and act as a source to repair the damage of their specific tissue; and induced pluripotent stem cells (iPSC), which are adult stem cells that are reprogrammed into another type of adult stem cells and one of the most important cells that can be used for medical purposes. Additionally, there are prenatal stem cells which come from the fetal membrane, umbilical cord, and amniotic fluid extra-embryonic cells, and mesenchymal stem cells (MSC) which are adult stem cells originating from bone marrow, liver, and muscles [2,3]. For a cell to be considered a stem cell, two criteria must be met. First, stem cells must possess an unlimited capacity for self-renewal in order to produce descendants that are identical to the original cell. Second, they must possess the ability to differentiate into other healthy specialized cells of the body [4]; this specialization can occur depending on the physiological needs of tissue and organs at different times [5].

These properties of stem cells make them highly valuable, especially for medical purposes. Much research on these cells has shown the potential usage of stem cells in treating many diseases, such as cancer [6,7,8]. Regenerative medicine is one of the therapeutic applications of stem cells which can help to restore damaged organs or tissues in patients suffering from chronic diseases or injuries [9]. Another application of stem cells is their use in testing new drugs prior to animal and human trials [10]. Today, with the advancement of technology, various tools have been provided to researchers, among which we can mention genome editing techniques, which have given us the ability to change and manipulate the genome sequence at the desired point. By using this technology in stem cells, an important tool has been provided for researchers in the production of drugs and advanced treatments. In the following section, we introduce CRISPR, which is one of the most important tools in genome editing.

**Figure 1:**
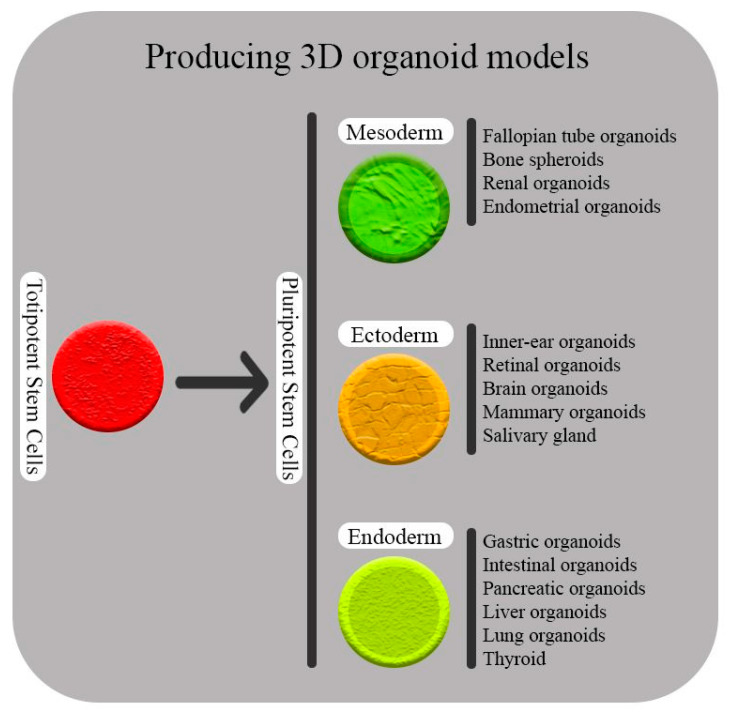
The zygote cell and its initial divisions (embryonic cells), which are considered totipotent stem cells, have the capacity to give rise to fully developed living organisms (body and placenta). Pluripotent stem cells, which can generate all cells that make up a live organism’s body following totipotent stem cells, are the next stage (mesoderm, endoderm, and ectoderm). Organoids are the new era for disease modeling, homeostasis, and development studies. Organoids derived from mesoderm, endoderm, and ectoderm are considered a new field of interest in biomedical research.

The cluster regulatory interspaced short palindromic repeats and CRISPR-associated protein 9 (CRISPR-Cas9) system consists of a short and repetitive nucleotide that was first discovered in the genomes of bacteria and archaea that act as adoptive immune systems. It works by removing exogenous genetic elements that assemble with Cas proteins [11]. The deactivation of the endogenous genetic elements consists of three steps. First, a few endogenous short nucleotides might integrate with the host’s CRISPR loci as new spacers. Then, a crRNA/Cas complex is created by the transcription of CRISPR RNAs (crRNAs). Finally, under the base complementation pairing rule, the complexes will inactivate the exogenous element [12]. Most of the RNase and DNase activities of Cas proteins are predicted with bioinformatics tools. New-generation sequencing has led to the discovery of a large number of Cas proteins; thus, new classification based on sequence information is necessary. The new classification is various and has evolved fast. The new classification has three categories: type 1, type 2 (Cas9 is included in this type and is based on the presence of the HNH domain), and type 3; each type contains a large number of Cas proteins. Despite this classification, some subtypes of Cas proteins are still unclassified. This issue would be improved by further studies in the topic [13,14]. The type II CRISPR-Cas9 immune system stands out among them because it uses RNase III for cleaving the transcript into mature crRNAs and only needs one Cas9 protein to form a crRNA/Cas9 complex. This technology is simple, fast, cheap, and applicable. These characteristics make it a strong candidate for the development of a completely new genome-editing tool for biological and medical research (explained in Section 2) [15]. Gene editing using CRISPR/Cas9 technology has been widely implemented in biological and biomedical research, and stem cell-mediated cell treatment and gene therapy are recognized as essential elements in human medicine because of their capacity for tissue repair and regeneration [16]. Some different stem cell types have so far been successfully used in clinical studies and have received scientific approval. Besides viral genome modification, more and more publications are confirming that CRISPR/Cas9 genome editing is a potent technique that can greatly advance biomedicine, such as in virus genome editing and in stem cell research [11,17,18,19]. Organoids are interesting new systems that help us improve our knowledge of diseases’ mechanisms, development, evolution, homeostasis, and therapy. They can derive from ESs, ASCs, and PSCs (or even differentiate cells) (Figure 1). Organoids form three-dimensional (3D) cell culture models (in vivo-like morphogenesis), and our understanding of signaling pathways (cascades) that result in this formation is important. Indeed, utilizing the CRISPR/Cas9 system for editing in organoids increases our understanding of different disorders (e.g., human digestive diseases). This is mostly through removal of genes of interest and investigating their role in such conditions. Genome-wide CRISPR screens are another applicable tool that help researchers to find mutations and genes involved in organ regeneration, tumorigenesis, metastasis mechanisms and off-target analyses (this topic is discussed in Section 2.2) [20,21,22].

### Stem Cell Application in Medicine

Stem cell therapy has the potential to cure a wide range of aggressive and deadly human diseases. Physiological, morphological, and developmental subjects, in addition to formation, regeneration, and repair of tissues have all benefited from stem cell research during the last decade [23,24,25]. Recent advancements in stem cell technology have allowed scientists to use ex vivo and in vivo stimulation to differentiate stem cells into functional offspring for therapeutic purposes [26,27]. Many studies have shown that particular stem cells can be used to treat a variety of human pathological conditions [28,29,30]. Additionally, engineered stem cells could be employed to cure and reverse inherited genetic abnormalities [31]. Stem cells have been found to be a promising therapeutic tool for treating pathological conditions like cancer by transferring altered genes to the injured organ/tissue [32,33]. NSCs, for instance, have been demonstrated to migrate via the central nervous system and reach the extra cranial neoplastic location [32,34,35]. Despite all the advantages of stem cells, there are several disadvantages that have limited their routine clinical use until now. Some of these limitations include their heterogenicity and instability (stem cell malignant transformation), long term immune rejection, and limited access to ASCs; these are some of the reasons which have resulted in few confirmations for their clinical application [36,37,38,39]. Different immunological behaviors were observed from stem cells derived from different tissues. For example, hiPSC-derived retinal pigment epithelial (RPE) cells were tolerated in different locations, but autologous integration-free hiPSCs have shown immune rejection [40]. In another example, researchers noticed that fetal liver-derived MSCs are more immunosuppressive and immunoregulatory than bone marrow-derived MSCs [41]. Researchers have been attempting to circumvent these limitations by inventing and discovering new technologies such as genome editing tools like CRISPR-Cas9 and CRISPR/Cas12. By using these technologies, we can delete gene(s); insert or delete nucleotide(s), genes, or gene clusters; and inhibit or activate gene expression. Also we can manipulate RNAs with the CRISPR/Cas13 method [42]. This system has been used to evaluate the function of genes in stem cells and to optimize their function. In the following sections, these functions will be discussed in detail.

## 2. CRISPR/Cas

### 2.1. CRISPR/Cas9 and CRISPR/Cas12

CRISPR is a technique that is designed for targeted modification of specific DNA or RNA sequences; nevertheless, it sometimes produces unwanted or unexpected changes in DNA [43]. In bacteria and archaebacteria, this mechanism works as an adaptive defense against invading nucleic acids (phages) [44]. CRISPR/streptococcus pyogenes CRISPR associated protein 9 (spCas9), the most well-known CRISPR system, is derived from the Streptococcus pyogenes bacterium [45,46,47]. In Streptococcus pyogenes, this system is made up of two primary components. An RNA (containing two distinct RNAs named CRISPR RNA (crRNA) and trans-activator RNA (tracrRNA)) and the Cas9 endonuclease protein, which targets crRNA and tracrRNA [48].

Bioinformatics methods were used to create the functional system-related RNA of this bacterium, and instead of two independent components (crRNA and tracrRNA), it has become a single guide RNA (sgRNA). The 20 nucleotides in terminal 5’ of the sgRNA are designed to complement the DNA target location. The presence of a sequence called protospacer adjacent motif (PAM), consisting of 5′-NGG-3′, on the target sites at the end of three of these 20 nucleotides is required for system function [49,50]. In terms of length and nucleotides, this sequence differs amongst bacteria [51,52,53].

**Figure 2:**
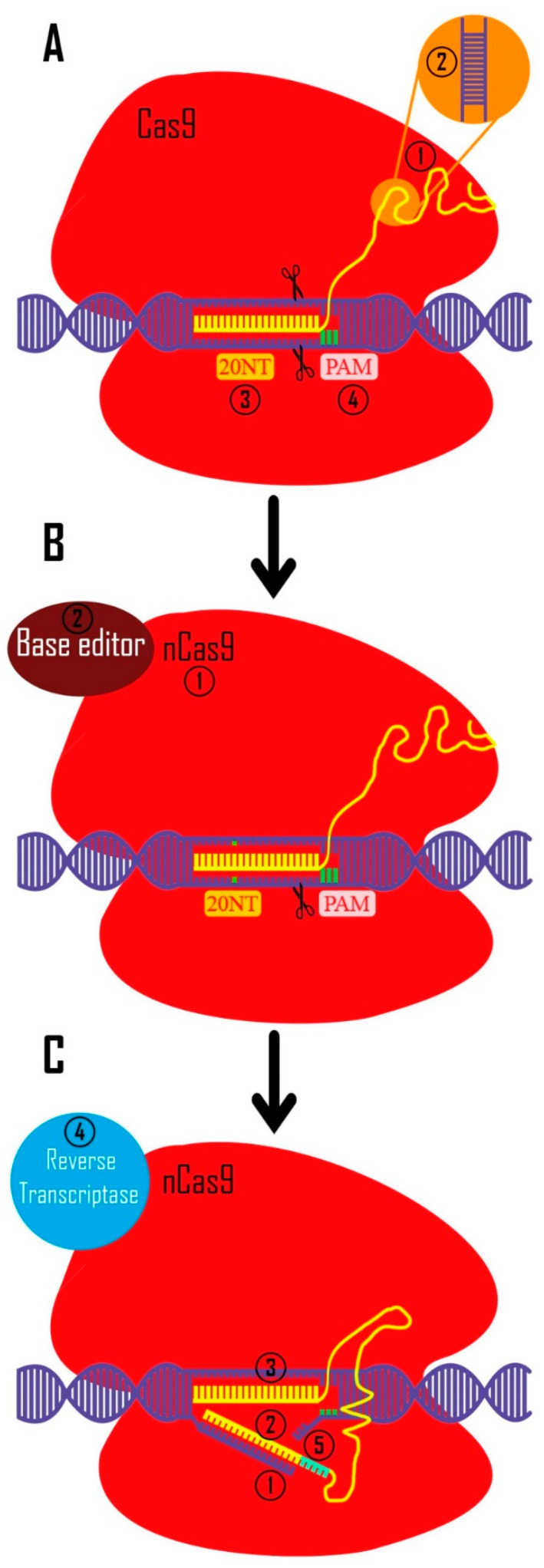
CRISPR toolkit. (**A**). CRISPR technology was originally used to create double-strand breaks in eukaryotic DNA (with a bacterial origin (Streptococcus pyogenes)). 1. In bacteria, crRNA and tracrRNA guide Cas9 to target the intended region. These RNAs are artificially synthetized as a unique sgRNA to be more applicable in other creatures (yellow) 2. crRNA and tracrRNA are widely used in multiple experimental systems (e.g., mouse embryo microinjections, RNP electroporation into mammalian cell lines, etc.) [91,92] 3. Twenty nucleotides complementary to the target site are used to identify the target area (these nucleotides are designed in a targeted manner). 4. Before these 20 nucleotides, there are three PAM nucleotides (5′-NGG-3′ in Streptococcus pyogenes Cas9 system) which are necessary for CRISPR/Cas9 function. (**B**). 1. In order to modify the bases in a targeted way, the Cas9 protein was altered to cut only one strand of DNA by changing one amino acid in Cas9 protein (nickase Cas9 [nCas9]). 2. Additionally, they coupled the different base editor domains to the Cas9 protein. (**C**). 1. Prime editing, the subsequent iteration of this technique, cuts a DNA strand by creating a cut at the intended location. 2 and 3. The sgRNA is made in such a way that its 3′-end complements the two sides of cut site, and its 5′-end can recognize the target site. 4. The reverse transcriptase enzyme turns 3′ sgRNA into cDNA using this 3′ end as a primer. 5. In the cut region, bases are designed for knock-in to produce highly accurate results.

The Cas9 protein identifies this region after the 5’ sgRNA ends of 20 nucleotides bind to the target site. The Cas9 protein then makes a blunt end double-strand break in DNA [51,52,53]. It can take one of two paths after breaking the DNA code—the cell either commits suicide or fixes the damage (Figure 2) [54]. NHEJ (non-homologous end joining) and HDR (homology directed repair) are the two main processes for repairing double-stranded breaks in DNA [55]. In mammals, the NHEJ mechanism is the most common mode of repair, and most cells use it to mend double-stranded breaks. A few nucleotides are inserted/removed, or maybe larger deletions occur quite often. Moreover, in certain reports, the loss of chromosome arms or deletions of thousands of kb have been reported in the cut region during NHEJ repair (INDEL mutation) [56,57]. A frameshift mutation occurs when the insertion and deletion of nucleotides do not multiply by three, resulting in loss of gene function (knock-out) [58]. The capacity to target several DNA loci concurrently (multiplexing) is one of the system’s features [59]. The knock-out method has been widely utilized to assess the function of genes involved in stem cell stemness and differentiation. Using this technology, the histopathological role of mutations and diseases mechanisms were assessed with the help of stem cells. For example, paired-like homeodomain 2 (PITX2) (a transcription factor involved in atrial fibrillation (AF) disease), beta-2-microglobulin (B2M) (a serum protein that is associated with human leukocyte antigen (HLA) class I), and galactosidase alpha (GLA) (to produce a cell line model for Fabry disease (an X-linked inherited disease)) which is related to polysaccharides, glycoproteins, and glycolipids cleavage, were targeted to find and model the roles of those genes in development of disease mechanisms within the stem cells, which in turn provided new insights for designing new therapeutic approaches [60,61,62,63]. Some of the other studies are addressed in Table 1. CRISPR/Cas12 is another form of this system. It was first discovered in Acidaminococcus and Lachnospiraceae bacteria, and afterwards in additional bacteria in various forms. Double-strand sticky end breaks in DNA, DNA cut distance from PAM, longer binding-site-to-target region, shorter sgRNA length, smaller Cas protein size than CRISPR/Cas9, and AT-rich PAM (unlike CRISPR/Cas9) are all features of this system [49,64,65]. A large number of Cas12 (Cas12a to k) have been identified so far [66], but the most studied and practical system for stem cells is still Cas12a. Base editing (BE) and prime editing (PE) represent the next generation of CRISPR technology. Using BE, we can change single nucleotides (in RNAs (temporary) or DNAs (permanent)). If BE efficiency, precision, and specificity are improved, it has high potential for being used in personalized medicine [67]. PE is one of the latest technologies derived from Cas9. Modifications have been applied to this technology to create changes in DNA that have the highest efficiency and are the least off-target. Several generations of PEs are being developed. The latest generations, PE4 and PE5, have two-fold increased efficiency compared to the previous one (PE3). In these generations (PE4 and PE5), an inhibitory mismatch repair (MMR) protein has been added to the Cas9 nickase protein and it has proper editing efficiency in-vitro [68]. Furthermore, many changes are being made to increase the efficiency of this technology. For example, by adding different domains to the Cas9 nickase protein and making changes in peg RNA, the efficiency of this technology has been greatly improved [69,70,71]. Spinal muscular atrophy (SMA) is an autosomal recessive disease that is caused by mutations in SMN-2 and SMN-1 genes and causes problems in motor neurons. Mutations in the SMN-1 gene are fatal to fetuses, but adults who have mutations in the SMN-1 gene have the disease. Researchers removed intronic splicing silencer-N1 (ISS-N1) by PE technology in SMA-patient specific iPSCs (SMA-iPSCs) (24/7 efficiency) [72]. In another study, editing hiPSC-derived cardiomyocytes of Moloney murine leukemia virus with split and non-split reverse transcriptase (RT) deleted the RNase H domain; MMLV-RTΔRH showed 1.4% to 16.7% editing efficiency in all four locations [73].

HDR repair is used much less in human cells. DNA is employed as a template in this sort of repair, and the repair is precise. As a result, the repaired DNA will be identical to the original DNA. With this repair we can change nucleotide(s) in the target DNA, or nucleotides/gene fragments can insert into this region utilizing this method of repair (knock-in) [89,90].

### 2.2. Genome-Wide CRISPR/Cas Knock-Out

The genome-scale CRISPR knock-out (GeCKO) method was first developed to examine genes in the human genome [93]. With the addition of non-coding areas as new targets for the CRISPR library system, the technology was improved to GeCKO V2 (GeCKO Version 2) [94]. This method (genome-wide screening) has been frequently utilized to identify key and fundamental genes involved in diseases [95,96]. This technique was employed in a study assessing loss of function in iPSC-derived macrophages. The function of macrophages in chronic inflammation, neurological disorders, cancer progression, and immune response was revealed for the first time in this study [97]. Another study on ESCs looked at metabolic signals for transitions in embryonic cell fate and discovered that inhibiting the Tfe3 gene causes ESC differentiation [98]. This method was also utilized to explore the medication resistance of pancreatic cancer stem cells, which led to the recommendation of autoimmune medicines as a novel treatment option for pancreatic cancer patients [99].

### 2.3. Dead/Deactivated Cas9 (CRISPR i or a (Inhibition/Activation))

Researchers discovered that D10 (aspartic acid 10) in the RuvC domain and H840 (histidine 840) in the HNH domain are the two amino acids responsible for Cas9 protein double strand DNA cleavage [49]. The Cas9 protein loses its capacity to create cuts in the target region when certain amino acids (D10A (aspartic acid 10 alanine) and H840A (histidine 840 alanine)) are changed. Dead/deactivated Cas9 (dCas9) is a modified protein that can only connect to the target site (directed by sgRNA) [100]. This protein guides functional domains to the target region chosen by sgRNA by transposing protein functional domains into dCas9. For example, to increase and reduce mRNA expression, dCas9-binding transcription-activator and inhibitor domains are utilized (CRISPR a and I (CRISPR activator and inhibitor)). The sgRNA was created with transcriptional regulatory areas in mind. The functional domain of raising or reducing mRNA transcription is transferred to this area by dCas9. In fact, dCas9 is a carrier of specific functional domains [101,102,103] (e.g., epigenetic regulatory protein domains (acetylase, methylase, deacetylase, demethylase) [104], green fluorescence protein (GFP) to identify the DNA locus in the cell [105], and base editors to modify the nucleotide without cutting in DNA (Figure 2) [106]) and has proved to be a powerful tool of this technology.

This technology has been widely utilized to investigate the impact of changing gene expression levels on stem cell activity. In mouse ESCs, dCas9-Kruppel associated box (KRAB) inducible with doxycycline knock-in was used to study gene expression and repression, cell differentiation, or reprogramming [107]. In another investigation, in MSCs, CRISPR/dCas9-KRAB (CRISPRi) and CRISPR/dCas9- *herpes simplex virus*-based transcriptional activator *VP64* domain (VP64) (CRISPRa) inducible with doxycycline (it has an inducible promoter that becomes activated with doxycycline) were used to control the expression of alkaline phosphatase (ALP) gene. In this setting, the osteoblast differentiation capacity of MSCs was enhanced or inhibited in vitro [108]. Multiple activation with CRISPRa in adipose stem cells leads to the expression and production of nerve growth factor (NGF), brain-derived neurotrophic factor (BDNF), and glial cell line-derived neurotrophic factor (GDNF). Designed sheets with NGF, BDNF and GDNF neurotrophic factors related to gene activation in the rats induced Schwann cells in vitro to improve migration, proliferation, and neurite extension and promoted nerve reinnervation, regeneration, and efficient in vivo recovery [109]. Table 2 has more examples of this platform application.

### 2.4. RNA Editing

Another kind of this system is CRISPR/Cas13. The only difference is that it breaks RNA rather than DNA [113]. Cas13a, b, c, and d are four different types of this system, each with its own set of characteristics [113,114,115]. In CRISPR/Cas13, the area known as PAM in CRISPR/Cas9 and CRISPR/Cas12 is termed as PFS (protospacer flanking site) [115]. However, other Cas13 varieties (such as Cas13d) do not require PFS [116]. In RNA, this mechanism causes a break [117]. Changes to the amino acids in the Cas13 protein have been made, similarly to the CRISPR/Cas9 and CRISPR/Cas12 systems, so that the Cas13 protein binds to the target site only with crRNA guidance. dCas13 gains the ability to effect modifications at the RNA level by binding particular functional domains [118,119,120]. In one study, scientists created an exon-specific isoform expression reporter system (EXSISERS) to determine the type of isoform expressed in cells. With this method, a link between protein and polypeptide is established by intein (a protein that mediates the split-Cas9 system) without affecting the protein, and it is efficient for detection of RNA isoforms. This method was used to evaluate exon 10 of tau protein in iPSCs derived from a patient and the effect of targeting effectors for the specificity of isoforms by use of Cas13. This showed that the method was sensitive and applicable for this purpose [121]. 

### 2.5. Off-Target

Dedicated sgRNA design is normally carried out using web-based applications [122,123,124]. The most crucial component of a basic design is selecting the optimal sgRNA, created by the software, that has the maximum performance and specificity. Following design, sgRNAs are examined for practical performance in in vitro and in vivo settings, as well as the ability to make cuts (INDEL mutations) using methods such as next-generation sequencing, T7E1, or SURVEYOR kits [125,126]. Once the best sgRNA has been determined, it will be used for further research. The risk of sgRNA binding to places other than the target site and producing cuts in those regions (off-target) is one of the issues with the CRISPR system [127]. Various approaches are employed to prevent these off-target cuts. Double nickase is one of these techniques. The method employs Cas9 systems that have been engineered to cleave a strand of DNA. One dCas9 cuts the sense strand, and the second dCas9 cuts the antisense strand at the same time, resulting in a sticky end double-strand break [128]. The off-target cuts are reduced by 50 to 1500 times using this strategy [50]. This technique has been utilized to limit the generation of off-target stem cells. R201H has been reported to increase intracellular cGMP production in hPSCs cells utilizing this technology in the guanine nucleotide-binding protein alpha gene stimulating activity polypeptide 1 (GNAS) gene. These cells can be utilized to figure out how the GNAS gene works [129]. To test the function of the genes RB1 (retinoblastoma 1) and immune-reactive antigen domain containing 1 (OCIAD1) in iPSCs, this method was employed to generate heterozygous knock-out iPSCs [130,131].

Other approaches include shortening the sgRNA target region to 17 nucleotides to lower the chance of mismatches, sgRNA and Cas9 protein engineering, and the use of particular medicines [132,133,134]. In human stem cells however, off-target mutations are rare [135].

### 2.6. Knock-In

The knock-in method can be accomplished by a number of ways. All of these techniques are based on using donor DNA to make nucleotide modifications or add a gene or gene cluster to the target site [136]. A DNA fragment with a length of 100 to 200 bp can be used to make modifications in several bases. The changed nucleotides are placed in the ssODN middle where the DNA is cut, and the 3′ and 5′ sides of these single stranded oligodeoxynucleotides (ssODNs) are complementary to the two sides of the CRISPR system’s target site. HDR repair is accomplished after DNA cleavage, and the desired nucleotides are altered [137,138]. Another option is to use prime editing to make these changes. The reverse transcriptase enzyme is coupled to the nickase Cas (nCas) protein in this approach. The sgRNA is likewise built so that its 5′ end is complemented by two sides of the cut area, with the nucleotides we want to modify placed in the middle (at the cut site).

A 5′-strand sgRNA complement is inserted into the target site once the sgRNA is bound to the target area (the DNA acts as a primer, and the reverse transcriptase enzyme synthesizes DNA from the RNA). This DNA serves as a donor template for the cell. The HDR repair system repairs the target spot, and KI occurs with great accuracy (Figure 2) [139].

We can use longer homology arms on either side of the insert site to insert chunks longer than the donor DNA [140,141]. Drugs with different methods (e.g., Nocodazole (G2 and M phase cell cycle arrest), RS1 (binding homologous recombination-binding protein (RAD51) to the cut DNA area facilitators), and nu7441 molecules (NHEJ inhibitor)) considerably boost HDR repair and hence KI efficiency [142]. They monitor long-term expression changes by attaching reporter genes (KI) to the c-terminus of stemness-related genes [143,144,145,146]. In Wilson’s disease hiPSCs, the mutation in R778L (arginine 778 lysine) in ATPase copper transporting beta (ATP7B) was induced; this has application in drug screening and finding disease model mechanisms for Wilson disease [147]. In iPSCs derived from a patient with global development delay, the c.1730T>A mutation was induced in the mental retardation autosomal dominant 7 (MRD7) gene [148]. In iPSCs-derived motor neurons amyotrophic lateral sclerosis, the G4C2 hexanucleotide repeat expansion (HRE) was replaced with a normal region (HRE complete correction) by CRISPR-Cas9 and homology-directed repair [149]. Urinary-induced iPSC-derived monomeric cardiomyocytes red fluorescence protein, firefly luciferase (Fluc) for bioluminescence and herpes simplex virus, thymidine kinase for positron emission tomography (PET) imaging Cells imaging, approved these cells’ enhanced cardiac function in infarcted heart [150]. Table 3 shows examples of knock-in and its uses in stem cells.

## 3. CRISPR Delivery Methods

Delivery methods are classified into two categories: viral vectors and non-viral vectors [158]. Safety, low immunogenicity, specific function, minimal toxicity, and high efficiency are all critical properties of a good vector [159,160]. Adenoviruses, adeno-associated viruses (AAVs), and lentiviruses are the most common viral vectors. These viruses are being used in a number of clinical investigations [159]. Lentiviral vectors can transport larger amounts of DNA, and the third generation of these viruses is undergoing clinical trials [161,162]. Additionally, these viruses have the ability to penetrate cells efficiently, produce a large number of in vitro viruses, and they have a high cell transduction rate [163,164,165]. However, one of the most essential characteristics of viruses is their capacity to integrate into host DNA. AAVs were found, in a 10-year long-term study, to have the ability to integrate into DNA and induce cancer in dogs with hemophilia who have been treated with the virus [166]. The virus can enter the genome through the CRISPR system’s cut area and remain there for a long time [167,168]. To overcome this problem, virus-like particles that do not have the ability to integrate their genome into the host’s genome are used. This delivery method is used for both in vitro and in vivo editing. These viruses are non-proliferative and have good delivery efficiency [169]. The PiggyBac transposon system is the other technology for gene(s) or cluster delivery. By this technology, dCas9-VPR and dCas9-KRAB transgenes and sgRNAs are delivered to hPSCs to change TCF4 gene expression (a transcription factor for NSCs regulation). Data has shown that this method is capable of stable delivery [170]. 

Immunogenicity is another issue with these viruses. The immune system responds to these viruses in the early stage of their admission into the body, and the body develops immunity to their re-entry [171].

Mechanical procedures such as electroporation and microinjection are examples of non-viral carriers [172,173]. Liposomes and nanoparticles are the two primary kinds of chemical techniques (metal nanoparticles and lipid nanoparticles). These approaches are generally safe, have a low immunogenicity, and are primarily employed in ex vivo studies [174,175,176].

## 4. Conclusions

CRISPR-based stem cell genome editing is a novel field in regenerative medicine. This technology has the ability to make precise changes in DNA and RNA that could ameliorate stem cell therapy. Knocking-out, knocking-in, base editing, and changing RNA expression are all some of the possibilities with this technology. Researchers employed this technique to examine and improve stem cell function by better understanding the involvement of important genes and biological cascades. This technology is expected to dramatically expand our understanding of stem cells and facilitate the use of these cells in therapy.

## Figures and Tables

**Table 1:** Application of CRISPR knock-out system in studying the function of stem cell genes (all of the knock-outs done by CRISPR-Cas9 system).

Stem Cell	Gene(s) Knock-Out	Gene Function	Results
Pancreatic cancer stem cell	PolymeraseII-associated factor 1 (PAF1)	Regulates stem cell features (i.e., decreased the orthotopic pancreatic tumors capacity to expand and evolution in mice and cancer stem cells)	The PAF1 interaction with PHF5A (PHD Finger Protein 5A), DDX3 (DEAD-box RNA helicase 3) genes to regulate stemness with NANOG expression [74].
Mouse Embryonic stem cell	Recombination activating 1 gene (RAG-1)	Associated in immunoglobulin V-D-J recombination activation	Efficiently create RAG1 biallelic homozygous and compound heterozygous indel mutations (92%) [75].
hESCs	Fos Proto-Oncogene, AP-1 Transcription Factor Subunit (FOS)	Involved in cell differentiation, proliferation and transformation regulator	HESCs line production for hematopoietic differentiation assay [76].
Pluripotent stem cell	WW Domain Containing Transcription Regulator 1 (WWTR1)	Associated with signal transduction, differentiation, and hippo signaling regulation	WWTR1 knock-out does not have any effect on karyotype, phenotype, and differentiation [77].
hESCs	RAP1 (Ras-proximate-1)	A small cytosolic GTPase that is vital for signal transduction	RAP1 deficiency enhance self-renewal and delay cellular senescence. Therefore, It has a role in hESCs homeostasis (telomeric and non-telomeric role) [78].
hESCs	Histone deacetylase 6 (HDAC6)	A transcription repressor	HDAC6 homozygote knockout in hESCs does not have any effect on karyotype, differentiation, and pluripotency [79].
Spermatogonia stem cell	EPH receptor B2 (Ephb2)	Tyrosine kinase receptor that has role in differentiation, division and motility	The Ephb2 knockout cells showed less colonies compared to the wild type cells, which demonstrated the role of this gene in pluripotency [80].
hESCs	Poly (ADP-Ribose) Polymerase 1 (PARP1)	A chromatin-associated enzyme that has associated with tumor transformation, proliferation, differentiation and cell damage recovery	PARP1 knock-out cell lines showed normal differentiation ability, karyotype and stem cell markers expression [81].
mESCs	The Methyltransferase-like 3 and 14 (METTL3, METTL14)	Complex of methyltransferase that are sequence-specific DNA adenine methyltransferase (in unpaired and single-strand DNA)	m6 A RNA methylation as a way to restrict ERVs [82].
hMSCs	NAD-dependent deacetylase sirtuin-3 (SIRT3)	Is a histone deacetylases that has widespread effects in nuclear gene expression control	SIRT3 knock-out resulted in the detachment of genomic lamina-associated domains (LADs) from the nuclear lamina, chromatin accessibility increases and enhance cell senescence [83].
hMSCs	Receptor activator of nuclear factor kappa-Β ligand (RANKL)	An apoptosis regulator that is involved in immune system, bone remodeling, regeneration and controls cell proliferation	The mesenchymal stem cells showing capacity of bone formation is immortalized [84].
mESCs	Telomeric repeat binding factor 2 (TRF2)	A key gene for telomeres protectection	TRF2 knock out showed that it is dispensable for the proliferation and survival of mouse embryonic stem cells [85].
Myeloma cells	V-Set Pre-B Cell Surrogate Light Chain 1 (VPREB1)	Involved in early stages of B cell development	Knock out of VPREB1 effective in inhibition of primary myeloma grows [86].
hESCs	Acidic nuclear phosphoprotein 32 family member A (ANP32A)	A RNA binding protein that is associated with nucleocytoplasmic transport	The knock out cells shows the normal karyotype and typical stem cell morphology, in accordance with high expression of pluripotent genes and the differentiation potential in-vitro [87].
Adult epithelial stem cells	Interferon Regulatory Factor 2 (IRF2)	An interferon regulatory factor	IRF2 is an antagonist of stemness. With the knock-down of this gene in Keratinocytes, migration, self-renewal and epidermis formation increases [88].

**Table 2:** The effect of CRISPR a/i on the gene expression regulation in the stem cell assay.

Stem Cell	CRISPR System	Result
Adult epithelial stem cells	Interferon Regulatory Factor 2 (IRF2)	
Rat bone marrow-derived mesenchymal stem cell (rBMSC)	Endogenous SOX9 activation/peroxisome proliferator-activated receptor gamma (PPAR-γ) repression (dCas9 by modules with MS2 coat protein [MCP]-p65- heat shock factor 1 [HSF1] (MPH) as activation complex and Com-(Krüppel-associated box) KRAB (CK) as repression complex used fot this study)	This system in 2D culture arouses chondrogenesis and suppressed adipogenesis. However, the formation of manipulated cartilage and recovery of calvarial bone healing are enhanced in 3D culture system [110].
hiPSCs derived from Parkinson disease	CRISPR/dCas9-DNA-methylation (DNA methyltransferase 3A [DNMT3A])	CRISPR/dCas9-DNA-methylation (DNMT3A) designed for alpha-synuclein gene (SNCA) intron 1. As a result, 30% decrease in SNCA mRNA and protein expression were observed [111].
iPSCs	dCas9- *KRAB* repressor	Identify genes that differentiate iPSCs into Cardiomyocyte [112].

**Table 3:** Knock-in and application of this technology in stem cells (all the knock-ins done by using CRISPR/Cas9 technology).

Stem Cell	Gene	Result
hiPSCs	Pancreatic And Duodenal Homeobox 1 (PDX1) [a transcription activator for several genes]	EGFP receptor introduced in PDX1 c-terminal gene by CRISPR/Cas9 knock-in in KSCBi005-A-3 which is used to monitor PDX1 expression during B-cell differentiation in live cells [151].
hiPSCs	Activity-regulated cytoskeleton-associated light inducible (Arc light) [a kind of genetically-encoded voltage indicators]	Arc light stable expression from Adeno-Associated Virus Integration Site 1 (AAVS1) locus. This hiPSCs is useful for cardiac development studies [145].
Ovarian cancer stem cells	Green fluorescence protein (GFP) [a fluorescent protein]	GFP knock-in in NANOG gene is performed to study NANOG and androgen receptor (AR) expression and co-localization [152].
Recessive dystrophic epidermolysis bullosa-specific-iPSCs	Drug preserve selection cassette (i.e., a drug-resistance gene, for positive cells selection)	Properly corrected a pathogenic mutation in Autosomal recessive dystrophic EB (RDEB)-specific iPSCs [153].
iPSCs	Tetratricopeptide repeat domain 3 (TTC3) [ubiquitin-dependent protein catabolic mechanism and associated in protein K48-linked ubiquitination]	A cell line (p.S1038C) is used for the risk of late onset Alzheimer’s assessment [154].
hESCs	Akaluc (a sensitive luciferase reporter)	Insert Akaluc into the AAVS1 locus to generate human embryonic stem cell lines capable of being traceable with near-infrared emission light [155].
ESCs	GFI1-tag	Generating cells with GFI1-tag that can be identified via western blot and immunohistochemistry [156].
hESCs	RYBP (RING1 And YY1 Binding Protein) gene with Flag-HA	Normal morphology and karyotype, while it maintains pluripotency to differentiate into three germ layers [157].
